# Fermented liquid feed for pigs: an ancient technique for the future

**DOI:** 10.1186/2049-1891-6-4

**Published:** 2015-01-20

**Authors:** Joris AM Missotten, Joris Michiels, Jeroen Degroote, Stefaan De Smet

**Affiliations:** Laboratory for Animal Nutrition and Animal Product Quality, Department of Animal Production, Ghent University, Ghent, Belgium; Department of Applied Biosciences, Ghent University, Valentin Vaerwyckweg 1, B-9000 Ghent, Belgium

**Keywords:** Fermented liquid feed, *Lactobacillus* spp, Pigs, Probiotics, Yeasts

## Abstract

Fermented liquid feed is feed that has been mixed with water at a ratio ranging from 1:1.5 to 1:4. By mixing with water, lactic acid bacteria and yeasts naturally occurring in the feed proliferate and produce lactic acid, acetic acid and ethanol which reduces the pH of the mixture. This reduction in pH inhibits pathogenic organisms from developing in the feed. In addition, when this low pH mixture is fed, it reduces the pH in the stomach of pigs and prevents the proliferation of pathogens such as coliforms and *Salmonella* in the gastrointestinal tract. For piglets, the use of fermented liquid feed offers the possibility of simultaneously providing feed and water, which may facilitate an easier transition from sow’s milk to solid feed. Secondly, offering properly produced fermented liquid feed may strengthen the role of the stomach as the first line of defense against possible pathogenic infections by lowering the pH in the gastrointestinal tract thereby helping to exclude enteropathogens. Finally, feeding fermented liquid feed to pigs has been shown to improve the performance of suckling pigs, weaner pigs and growing-finishing pigs. In this review, current knowledge about the use of fermented liquid feed in pig diets will be discussed. This will include a discussion of the desirable properties of fermented liquid feed and factors affecting fermentation. In addition, advantages and disadvantages of fermented liquid feed will be discussed including its effects on gastrointestinal health, intestinal pH and the types of bacteria found in the gastrointestinal tract as well as the effects of fermented liquid feeds on pig performance.

## Introduction

Liquid feeding involves the use of a diet prepared either from a mixture of liquid food industry by-products and conventional dry materials, or from dry raw materials mixed with water. By definition fermented liquid feed is feed that has been mixed with water, at a ratio ranging from 1:1.5 to 1:4, for a period long enough to reach steady state conditions. If there is almost no time between mixing and feeding or the period for fermentation is too short to reach steady state conditions, the term liquid feed or non-fermented liquid feed is used [1].

By mixing with water, lactic acid bacteria and yeasts naturally occurring in various feed ingredients proliferate and produce lactic acid, acetic acid and ethanol which reduces the pH of the mixture [2]. This reduction in pH inhibits pathogenic organisms from developing in the feed [3]. In addition, when this low pH mixture is fed, it reduces the pH in the stomach of pigs and prevents the proliferation of pathogens such as coliforms and *Salmonella* from developing in the gastrointestinal tract [2].

The interest in the fermentation of feed for improving the performance of piglets and pigs increased dramatically after the announcement of the ban in the European Union on the use of antibiotics as antimicrobial growth promoters for swine. The potential of fermented liquid feed, as an alternative to the use of growth promoting antibiotics has been discussed in four recent reviews [1, 2, 4, 5]. In this review, recent information about the use of fermented liquid feed in pigs will be provided.

## Production of fermented liquid feed

Fermented liquid feed can be produced by fermenting a complete feed or by fermentation of the grain fraction and then mixing the fermented grain with other ingredients in order to formulate a complete diet [1]. Fermenting complete feeds is the easiest way to produce fermented liquid feed but this method can be associated with some problems. The fermentation process can cause a loss of essential nutrients such as vitamins and amino acids especially synthetic amino acids which may have been added to the feed [6–9]. Therefore, some authors advocate fermentation of the grain fraction only instead of the complete feed [7, 8, 10–14]. The fermented grain fraction may be used to make a range of diets, so that “phase feeding” can be implemented using the same fermented grain. Grains are also a more consistent product to ferment, compared with a complete feed containing multiple ingredients [8]. In addition, fermentation of cereals often results in a more rapid fermentation as cereals have a lower buffering capacity than compound feeds [2].

In order to successfully control the development of pathogenic organisms, fermented liquid feed must contain adequate amounts of lactic acid [15]. Lactic acid production can arise from spontaneous fermentation or by inoculating the feed with a culture of lactic acid bacteria prior to fermentation. Spontaneous fermentation is most often conducted using batch fermentation. In batch fermentation, the feed and water mixture is fermented without replacement of a portion of the fermented liquid feed [11]. The advantages of this system is that fermentation is easier to control and if undesirable fermentation occurs, it is only one batch of feed that is ruined [8, 16]. However, batch fermentation can take several days in order to produce a quality fermented liquid feed. In addition, under commercial farm conditions, it is difficult to run a batch feeding system because it is virtually impossible to clean and sterilize the system at every filling [4].

Beal et al. [17] concluded that spontaneous fermentation is not a reliable system to obtain a safe and palatable final product since variations in the pattern of fermentation occur. In addition, other studies have shown that uncontrolled/spontaneous fermentation results in higher concentrations of both acetic acid and biogenic amines which adversely affect the palatability of fermented liquid feed diets [8, 9]. Therefore, spontaneous fermentation is not advisable. However, should it be necessary to use spontaneous fermentation, the quality of spontaneously fermented liquid feed can be improved by the addition of copper to the fermentation medium which speeds up lactic acid production [18].

The quality of fermented liquid feed can also be improved by the inoculation of the feed with lactic acid bacteria that rapidly produce high concentrations of lactic acid [8, 19–21]. Inoculation is particularly valuable when fermenting only the grain fraction, considering that the production of lactic acid should be higher to compensate for the dilution and buffering effects of the other feed components when incorporated into a complete feed [7]. Bacterial strains to be used as inoculants for production must have a high capacity for lactic acid production and should be active against enteric pathogens [15]. Therefore, a considerable amount of research has been conducted to select beneficial strains of lactic acid bacteria for fermented liquid pig feed production [20, 21]. For example, Missotten et al. [21] tested 146 strains of bacteria for their ability to control *Salmonella*. Bacterial species often used for inoculating feed to produce fermented liquid feed are *Lactobacillus plantarum* and *Pediococcus* spp. [1].

Another technique for ensuring adequate production of lactic acid is a technique known as ‘back slopping’ [22]. In this technique, fresh feed and water are mixed with material from a previously successful fermentation which acts as an inoculum for the new mixture [23]. This allows for the gradual selection of lactic acid bacteria and an accelerated fermentation [23]. Compared with batch fermentation which takes several days to produce a quality fermented liquid feed, fermented feeds produced by back slopping can be fed within a few hours. However, Brooks [7] pointed out the possibility that this may result in the development of a microflora dominated by yeasts. Abundant yeast growth can have either negative or positive effects on the nutritive value of fermented feeds depending on the strains present.

Plumed-Ferrer et al. [24] showed that maintaining 25% residual liquid in the tank to inoculate the fresh liquid feed added to the tank was sufficient to maintain a proper fermentation. Moran et al. [12] found that there was no advantage to keeping more than 20% of the fermented wheat when performing fermentation. Therefore, although a residual retention of 50% is mostly commonly used, it seems that a lower proportion can be used with 20% being the lowest percentage which still ensures desirable feed characteristic when using back slopping.

## Factors affecting the quality of fermented liquid feed

**Figure 1:**
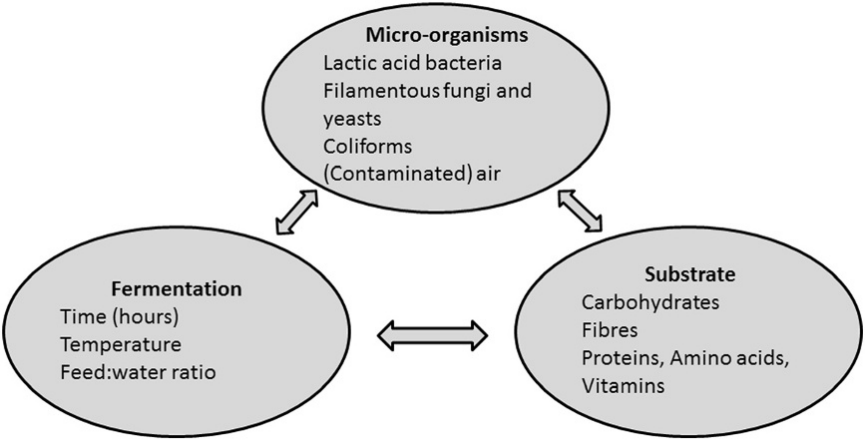
**Interactions in fermented liquid feed between the micro**-**organisms present**, **fermentation parameters and substrate quantity and quality affects the final end product**. Adapted from Niba et al. [26].

Factors affecting the quality of fermented liquid feed are displayed in Figure 1. Factors affecting the quality of the final end product include the types of micro-organisms initially present, substrate quantity and quality as well as various fermentation parameters [1, 2, 25, 26].

The amount of lactic acid bacteria naturally present on the feed or the amount of lactic acid bacteria added to the feed, determine the extent of lactic acid production. The faster this production, the faster the drop in pH and the faster pathogenic bacteria such as *Salmonella* spp. or *Escherichia coli* can be reduced [1].

In the past few years, studies have investigated the effects of population diversity of lactic acid bacteria or yeasts in fermented liquid feed [2, 13, 27–30], and a wide variation in the microbial population composition has been reported. *Lactobacillus plantarum* and *Pediococcus pentosaceus* tend to be the most abundant lactic acid bacteria strains present in fermented liquid feed [31].

Olstorpe et al. [31] reported that the composition of the bacterial species in fermented liquid feed changes during the fermentation process. They showed that *Pediococcus pentosaceus* was the dominant population at the beginning of a spontaneous fermentation, but after 3 days of continuous fermentation, *Lactobacillus plantarum* became the dominant population. This was also observed in inoculated fermented liquid feed where the lactic acid bacteria strain used to inoculate the feed did not remain the dominant lactic acid bacteria strain in the fermented liquid feed [1, 32].

The population diversity of yeasts present in fermented liquid feed is very high and deserves further investigation [29, 31]. In fermented liquid feed produced with wet wheat distillers’ grains, whey or tap water, the dominant yeast species tended to be *Pichia galeiformis*, *Pichia membranifaciens* and *Pichia anomala* respectively. In a more recent study, Olstorpe et al. [33] found another *Pichia* species, namely *Pichia fermentans*, to be the most abundant yeast species present, independent of the lactic acid bacteria culture used to inoculate the fermented liquid feed. However, Gori et al. [29] found that *Candida milleri* and *Kazachstania bulderi* were the predominant yeast species found in fermented liquid feed samples obtained from 40 Danish farms with an average contribution of 58.4 and 17.5% to the total yeast count.

The amount of yeast present can affect the quality of fermented liquid feed. Both positive and negative effects have been reported when the fermentation is dominated by yeasts depending on the stains of yeast present [1]. Yeasts have the ability of binding enterobacteria to their surface, thereby blocking the binding of these bacteria to the gut epithelium [34]. Therefore, high concentrations of yeasts in the fermented liquid feed may be beneficial. For example, Jensen and Mikkelsen [19] reported an inverse relationship between the concentration of yeast and enterobacteria in the gastrointestinal tract of pigs. In contrast, a high concentration of yeast can result in the production of “off-flavours” and taints due to the production of compounds such as acetic acid, ethanol and amylic alcohols which make the feed less palatable [8, 35].

Plumed-Ferrer and von Wright [36] indicated that the addition of weak acids during fermentation can successfully reduce the growth of yeasts without interfering with lactic acid bacteria development. Acids that showed good results were formic acid, potassium sorbate and benzoic acid. The addition of these acids may help to reduce problems (e.g. loss of energy, reduced palatability, foaming) resulting from excessive yeast growth. A drawback to yeast production can be the production of acetic acid, ‘off-flavours’ and ethanol, which may diminish the palatability as well as the dry matter and energy content of the feed [19].

Other parameters such as fermentation temperature, the interval between and the degree of back slopping (partial replacement of fermented liquid feed by fresh liquid feed in continuous fermentation) and the feed to water ratio used can also have an effect on the fermentation characteristics of the fermented liquid feed [8].

The effect of different temperatures on the quality of fermented liquid feed was studied by Jensen and Mikkelsen [19]. They reported that fermentation of feed at temperatures above 20°C did not provide any advantage over producing fermented liquid feed at 20°C. At 20°C, the coliform count was barely above the detection limit of 3 log_10_ CFU/g fermented liquid feed. However, the authors did stress that the temperature needs to be at least 20°C if the required pH at feeding is to be lower than 4.5. This is because enteric pathogens, such as *E. coli* and *Salmonella* spp., do not tolerate pH values below 4.5 [37].

Beal et al. [38] studied the effect of fermentation temperature on the exclusion of *Salmonella typhimurium*. Their results indicated that the time required for reduction of these bacteria was much shorter at 30°C compared with 20°C. Therefore, although the minimal temperature for obtaining optimal fermented liquid feed is a temperature of 20°C, a temperature of 30°C is preferable since it allows a more rapid production of lactic acid and a more rapid exclusion of any enteropathogens [16].

Adding cold water to the system should also be avoided with back slopping. For example, adding water immediately from the tap (5-7°C) will cold-shock the system. This could cause the induction of cold-shock protein formation in enteropathogens and this can protect them and allow them to persist for a longer duration in the feed [38, 39]. Furthermore, cold-shock inhibits the growth of lactic acid bacteria and allows yeasts to become dominant [39].

The feed to water ratio used for the production of liquid feed or fermented liquid feed can fluctuate between 1:1.5 and 1:4. From the overview given by Plumed-Ferrer and Von Wright [4] and Niba et al. [25], it appears that the most common slurry given to pigs involves a feed to water ratio between 1:2 and 1:3.

## Desirable characteristics for fermented liquid feed

Van Winsen et al. [3] described the desirable characteristics for fermented liquid feed as having a pH below 4.5, lactic acid bacteria concentrations above 9 log_10_ CFU/mL, lactic acid concentrations above 150 mmol/L and acetic acid and ethanol concentrations below 40 and 0.8 mmol/L, respectively. Beal et al. [38] reported that in order to prevent the growth of *Salmonella* spp., liquid feed needs to contain at least 75 mmol/L of lactic acid. Beal et al. [38] and Brooks et al. [8] reported that in order to reduce the concentration of enterobacteria, the concentration of lactic acid should be higher than 100 mmol/L. This concentration of lactic acid can have a beneficial effect on feed intake, daily gain and feed efficiency [40].

Although Van Winsen et al. [3] set the upper limit of acetic acid at 40 mmol/L, other authors indicated that a acetic acid concentration above 30 mmol/L could already negatively affect the palatability of fermented liquid feed [7, 8, 16]. However, Canibe et al. [41] reported that piglets fed fermented liquid feed with added acetic acid at levels up to 120 mmol/L showed no negative effects on body weight gain.

## Effect of fermented liquid feed on the microbes in the gastrointestinal tract

The composition of the microbial population in the gastrointestinal tract can be altered by the use of fermented liquid feed. The most common change is an increase in the concentration of lactic acid bacteria particularly in the stomach and small intestine [6]. Moran et al. [12], reported that the ratio of lactic acid bacteria to coliform bacteria in the lower gut of the pigs weaned using fermented liquid feed was shifted in favour of lactic acid bacteria, while in piglets fed dried feed, this ratio was shifted in favour of the coliforms.

The magnitude of the change can be affected by the fermentation conditions. For example, Canibe and Jensen [6] found no differences in the number of lactic acid bacteria present in the distal small intestine of growing pigs when the gastro-intestinal content was incubated at 37°C (Table 1). However, at an incubation temperature of 20°C (same as production temperature for the fermented feed), the proportions of lactic acid bacteria in the stomach and distal small intestine were significantly higher in growing pigs fed fermented liquid feed compared with dried feed or liquid feed.

**Table 1:** **Microbial counts** [**log**_**10**_ **CFU**/**g sample**] **along the gastrointestinal tract of pigs fed either dry feed**, **liquid feed or fermented liquid feed** (**feed to water ratio 1**:**2.5**, **back slopping with 50**% **retention at 20**°**C**)

	Diet			
Segment	Dry feed	Liquid feed	Fermented liquid feed	***P***-value
Lactic acid bacteria (20°C)				
Stomach	<5.4 (3)^a^	7.9^b^	9.0^c^	<0.01
Distal small intestine	<6.3 (5)^a^	<6.5 (3)^a^	7.2^b^	0.01
Caecum	<6.0 (5)	<6.2 (2)	<6.6 (2)	0.21
Mid colon	<6.1 (5)	<6.3 (3)	<6.3 (4)	0.34
Lactic acid bacteria (37°C)				
Stomach	8.8	8.7	8.9	0.35
Distal small intestine	8.2	8.6	8.4	0.41
Caecum	8.7^ab^	9.0^a^	8.3^b^	0.04
Mid colon	9.2^a^	9.2^a^	8.5^b^	0.01
Enterobacteria				
Stomach	3.8^a^	5.7^b^	<3.2 (4)^c^	<0.01
Distal small intestine	5.5^a^	6.6^b^	<4.1 (3)^c^	<0.01
Caecum	5.9^a^	6.3^a^	5.0^b^	0.02
Mid colon	6.2^a^	6.6^a^	4.7^b^	<0.01
Yeasts (20°C)				
Stomach	<3.4 (2)^a^	3.7^a^	5.4^b^	<0.01
Distal small intestine	<3.4 (3)^a^	3.9^b^	7.0^c^	<0.01
Caecum	<3.2 (2)	<3.3 (1)	<5.1 (1)	0.07
Mid colon	<3.2 (3)^a^	<3.3( 1)^a^	<4.6 (1)^b^	0.03
Yeasts (37°C)				
Stomach	<3.3 (4)^a^	<3.6 (2)^a^	4.2^b^	0.03
Distal small intestine	<4.0 (3)	3.6	4.5	0.08
Caecum	<3.9 (2)	<3.4 (3)	<3.6 (3)	0.59
Mid colon	<3.7 (3)	<3.3 (4)	<3.4 (2)	0.69

Values in brackets indicate the number of samples with values below detection levels. The approximate detection levels (log_10_ cfu/g) were as follows: stomach: lactic acid bacteria, 5; enterobacteria, 3; yeasts, 3. Small intestine, caecum and colon: lactic acid bacteria, 6; enterobacteria, 4; yeasts, 3. “<” indicates that some observations from which the mean was calculated had values below detection levels. When no colonies were detected, the detection limit was applied to make the calculations. Therefore some values are lower than actually reported.

^a,b,c^Means within rows with a different superscripts are significantly different (*P* < 0.05).

Adapted from Canibe and Jensen [6].

Another significant change in the microbial population in the gastrointestinal tract is an increase in the number of yeast cells (see Table 1). As noted earlier, yeasts have the ability of binding enterobacteria to their surface, thereby blocking the binding of these bacteria to the gut epithelium [34].

The increase in lactic acid bacteria and yeast cells seems to be an excellent strategy to achieve a reduction of enteropathogens such as *Salmonella* spp. and *E. coli.* Recently, Canibe and Jensen [2] reviewed the value of fermented liquid feed in reducing enteric diseases in pigs. From surveillance studies, it is clear that fermented liquid feed reduced the incidence of *Salmonella* spp. [42–45].

## Effect of fermented liquid feed on pH in the gastrointestinal tract

The results obtained in a study by Canibe and Jensen [6] indicate the changes in pH in the different segments of the gastrointestinal tract when pigs are fed fermented liquid feed, liquid feed or dried feed (Table 2). The most dramatic change is a decrease in the pH in the stomach. The stomach is an important barrier against pathogens [46] and lowering the pH may strengthen this barrier and prevent coliform scours [47], especially in newly weaned piglets which are often incapable of producing sufficient amounts of gastric acid [48]. In addition, Radecki et al. [49] suggested that a lower gastric pH may allow better proteolytic activity in the stomach thus improving the growth of pigs fed diets containing fermented liquid feed.

**Table 2:** **The pH along the gastrointestinal tract of pigs fed either dry feed**, **liquid feed or fermented liquid feed** (**feed to water ratio 1**:**2.5**, **back slopping with 50**% **retention at 20**°**C**; **n** = **5**)

	Diet			
Segment	Dry feed	Liquid feed	Fermented liquid feed	***P***-value
Stomach	4.4^a^	4.6^a^	4.0^b^	<0.01
Proximal small intestine	5.9	5.8	5.7	0.48
Mid small intestine	6.0^a^	5.8^b^	6.1^a^	<0.01
Distal small intestine	6.4^a^	5.7^b^	6.1^ab^	0.02
Cecum	5.7	5.5	5.7	0.17
Proximal colon	5.9	5.8	5.8	0.72
Mid colon	6.1	6.0	6.1	0.54
Distal colon	6.4^ab^	6.2^a^	6.5^b^	0.04

^a,b^Means within rows with a different superscripts are significantly different (*P* < 0.05).

Adapted from Canibe and Jensen [6].

In contrast to the stomach, the pH in the small intestine of piglets fed fermented liquid feed is often higher than in piglets fed dried feed or liquid feed [6, 19, 50, 51]. This may be related to an increased secretion of pancreatic juice, stimulated by the low pH and high lactic acid concentrations in the fermented liquid feed [4, 19].

## Advantages of feeding fermented liquid feed

The principle benefit of feeding fermented liquid feed to pigs is that it improves performance. In this respect, Kil and Stein [5] have identified fermented liquid feed as one of the most effective feeding strategies to replace the use of antibiotic growth promotors. Beneficial effects have been observed with suckling pigs, weaner pigs and growing-finishing pigs. The magnitude of the improvement is related to the level of pathogens present in a given swine operation.

The new born pig has a sterile gut and acquires its characteristic flora through contact with its mother and the environment [52]. According to Kenny et al. [53], the period immediately after birth may be the most important window for establishing a potentially beneficial bacterial community, which can result in life-long, stable associations also called bacterial ‘imprinting’. Feeding sows fermented liquid feed influenced the bacterial gut population of their offspring [54]. Piglets from sows fed fermented liquid feed had lower coliform counts in their feces compared with piglets from sows fed non-fermented liquid feed or dry diets. In addition, the lactic acid bacteria counts were higher in the feces of piglets from sows fed fermented liquid feed compared with other piglets. This may be an indication that using the correct probiotic strain for producing the fermented liquid feed may result in microbial imprinting of the piglets’ microflora and therefore it may be possible to develop a bacterial population which is resistant to adverse ecological shifts at times like weaning.

Missotten et al. [1] presented a summary of several *in vivo* trials performed with dry feed, liquid feed or fermented liquid feed and their effect on the performance of weaner pigs. This confirmed the conclusions made earlier by Jensen and Mikkelsen [19]. In a summary of 4 trials comparing fermented liquid feed with dry feed they reported a 22.3% improvement in weight gain and a 10.9% improvement in feed efficiency.

A benefit associated with feeding diets in a liquid form is the fact that weaner pigs are provided with water and feed simultaneously [7, 39, 55, 56]. In this way, the piglets do not need separate learning for feeding and drinking behaviours [48, 55]. Barber [57] indicated that while some pigs may find a drinker within a few minutes of entering a pen, other pigs may take more than 24 h which is of a sufficient duration to induce symptoms of dehydration.

The results obtained by Russell et al. [55] demonstrate that the dry matter intake of the newly weaned pig can be increased by providing fermented liquid feed. When piglets are offered fermented liquid feed with different dry matter percentages (14.5 to 25.5%), they maintain their dry matter intake by increasing their total volumetric intake. The dry matter concentration of the diet also had no effect on weight gain or feed efficiency [58]. All of these studies support the theory that the pig will limit the intake of water not originating from liquid feed or fermented liquid feed (e.g. from nipple drinkers) to maximize feed intake [59]. Therefore, the total volumetric intake of dry matter and water will be comparable when the same diet is fed in liquid or dry form [58].

Since weaner pigs often have a higher dry matter intake when fed liquid feed or fermented liquid feed than when fed dry diets, when formulating diets to be used as fermented liquid feed, care should be taken to formulate on the basis of realistic estimates of dry matter intake. Otherwise, the piglets will consume too much of nutrients such as proteins which can depress feed utilization and ultimately depress dry matter intake [39] or cause protein-induced diarrhoea [7]. Brooks [7] pointed out that the fermentation of a nutritionally balanced feed will improve performance only if it increases feed intake or improves gut health. If intake is unaffected, it may well be that the biochemical changes produced by fermentation will produce a diet that is less nutritionally balanced.

The benefits obtained from feeding fermented liquid feed to growing-finishing pigs are not of the same magnitude as those obtained with weaner pigs [1]. Jensen and Mikkelsen [19] summarized the results of 9 *in vivo* trials comparing the performance of pigs fed dry feed and liquid feed and reported a 4.4% improvement in weight gain and a 6.9% improvement in feed efficiency with liquid feed. Although the improvements in performance obtained with growing-finishing pigs are not as great as those obtained with weaner pigs, there may be benefits in terms of carcass quality. Feeding fermented liquid feed has been shown to shift the conversion of tryptophan in the hind gut towards the production of indole instead of skatole resulting in a reduction in the concentration of skatole in the backfat of fattening boars and thus reduce boar taint [60]. Obviously, this benefit is only available under circumstances where intact males are used for finishing.

One explanation for the improvements in performance observed with fermented liquid feed is the control of pathogenic organisms [2]. However, another explanation may be an increase in nutrient digestibility. Although the results obtained when feeding fermented liquid feed are not straightforward, on average they seem to indicate a trend towards improved digestion [61–64]. This may be inherent to the fermentation processes, where there is a thin line between the formation of organic acids and activation of endogenous enzymes (e.g. phytase) in cereal grains which may increase digestibility and availability of certain nutrients [39, 65].

Fermentation of diets for 72 h (30-35°C) increased the ileal digestibility of crude protein, crude fibre and neutral detergent fiber and the total tract digestibility of crude protein in growing-finishing pigs [66]. One of the reasons suggested for the improved protein digestibility in pigs fed fermented liquid feed is related to the decrease in gastric pH [67]. A low gastric pH stimulates proteolytic activity in the stomach and slows the rate of gastric emptying which allows more time for digestion in the stomach to take place.

Significant improvements in the ileal digestibility of organic matter, nitrogen, and calcium have been reported in fermented liquid feed compared with dry feed [67]. A possible explanation for these increases is that feeding fermented liquid feed alters the morphology of the gastrointestinal tract [11]. Scholten et al. [11] reported that pigs fed fermented liquid feed had significantly greater villus length and a greater villus/crypt ratio, both characteristics that are associated with increased digestive capacity.

It has also been shown that fermentation of feed can cause mobilization of phosphorus from phytate by activation of endogenous grain phytase [67]. As a result, Lyberg et al. [67] reported a higher ileal digestibility of phosphorus in pigs fed fermented liquid feed compared with dry feed (30 vs. 48%).

Another advantage of fermenting feed is the possibility of reducing the content of various antinutritional factors contained in feeds [2]. Chiang et al. [68] fermented a rapeseed meal based diet and reported a 17% reduction in isothiocyanates after 1 day of fermentation and a 68% reduction after 3 days of fermentation. Fermentation of beans for 96 h reduced the concentration of antinutritional factors such as α-galactosides, phytate, trypsin inhibitor, tannins and saponins [69]. This was also seen in the study of Egounlety and Aworh [70] for fermentations of soybean, cowpea and groundbean. However, during the soybean fermentation the trypsin inhibitor increased slightly.

Reductions in the amount of dust in pig barns during handling and feeding have been reported with fermented liquid feeding [1]. Such a reduction not only improves the environment for pigs and workers but can help to exacerbate the impact of respiratory diseases on pig performance.

## Disadvantages of fermented liquid feeding

Although there are many advantages to the use of fermented liquid feed, there are also disadvantages. Liquid feeding is sometimes associated with the development of diseases such as haemorrhagic bowel syndrome, gastric torsion, gastrointestinal tympany and gastric ulcers [1, 7]. In addition, the fermentation process can cause a loss of essential nutrients from the feed especially synthetic amino acids deliberately added to the feed [6–9]. For example, the production of biogenic amines, such as cadaverine can occur as a result of decarboxylation of synthetic L-lysine, [8, 9]. Biogenic amine formation causes an irreversible loss of amino acids for the pig [71, 72]. The impact of this loss can be reduced by fermentation of the grain fraction only rather than the complete feed. Finally, if the feed is not properly fermented, a high concentration of yeast can result in the production of “off-flavours” and taints due to the production of compounds such as acetic acid, ethanol and amylic alcohols which make the feed less palatable [8, 37].

## Conclusions

Feeding fermented liquid feed to pigs has been shown to improve the performance of suckling pigs, weaner pigs and growing-finishing pigs. By reducing the pH in the stomach of pigs, feeding fermented liquid feed prevents the proliferation of pathogens such as coliforms and *Salmonella* from developing in the gastrointestinal tract. Additional benefits from liquid feeding include an increase in nutrient digestibility, improved intestinal morphology, a reduction in the content of various antinutritional factors in feeds and a reduction in dust levels in swine barns. However, liquid feeding is sometimes associated with the development of diseases such as haemorrhagic bowel syndrome, gastric torsion, gastrointestinal tympany and gastric ulcers. In addition, the fermentation process can cause a loss of essential nutrients from the feed especially synthetic amino acids. Finally, if the feed is not properly fermented, a high concentration of yeast can result in the production of “off-flavours” and taints due to the production of compounds such as acetic acid, ethanol and amylic alcohols which make the feed less palatable. On balance, the use of fermented liquid feed appears to be a cost effective alternative to the use of antibiotic growth promoters.
